# Impact of copper oxide nanomaterials on differentiated and undifferentiated Caco-2 intestinal epithelial cells; assessment of cytotoxicity, barrier integrity, cytokine production and nanomaterial penetration

**DOI:** 10.1186/s12989-017-0211-7

**Published:** 2017-08-23

**Authors:** Victor C. Ude, David M. Brown, Luca Viale, Nilesh Kanase, Vicki Stone, Helinor J. Johnston

**Affiliations:** 1 Nano Safety Research Group, School of Engineering and Physical Sciences, Institute of Biological Chemistry, Biophysics and Bioengineering, Heriot-Watt University, Edinburgh, EH14 4AS UK; 2 CNR-ISTEC Faenza, Via Granarolo, 64 -, 48018 Faenza, RA Italy

**Keywords:** Copper oxide nanomaterials, Caco-2, Toxicity, Interleukin-8, TEER, Translocation

## Abstract

**Background:**

Copper oxide nanomaterials (CuO NMs) are exploited in a diverse array of products including antimicrobials, inks, cosmetics, textiles and food contact materials. There is therefore a need to assess the toxicity of CuO NMs to the gastrointestinal (GI) tract since exposure could occur via direct oral ingestion, mucocillary clearance (following inhalation) or hand to mouth contact.

**Methods:**

Undifferentiated Caco-2 intestinal cells were exposed to CuO NMs (10 nm) at concentrations ranging from 0.37 to 78.13 μg/cm^2^ Cu (equivalent to 1.95 to 250 μg/ml) and cell viability assessed 24 h post exposure using the alamar blue assay. The benchmark dose (BMD 20), determined using PROAST software, was identified as 4.44 μg/cm^2^ for CuO NMs, and 4.25 μg/cm^2^ for copper sulphate (CuSO_4_), which informed the selection of concentrations for further studies. The differentiation status of cells and the impact of CuO NMs and CuSO_4_ on the integrity of the differentiated Caco-2 cell monolayer were assessed by measurement of trans-epithelial electrical resistance (TEER), staining for Zonula occludens-1 (ZO-1) and imaging of cell morphology using scanning electron microscopy (SEM). The impact of CuO NMs and CuSO_4_ on the viability of differentiated cells was performed via assessment of cell number (DAPI staining), and visualisation of cell morphology (light microscopy). Interleukin-8 (IL-8) production by undifferentiated and differentiated Caco-2 cells following exposure to CuO NMs and CuSO_4_ was determined using an ELISA. The copper concentration in the cell lysate, apical and basolateral compartments were measured with Inductive Coupled Plasma Optical Emission Spectrometry (ICP-OES) and used to calculate the apparent permeability coefficient (P_app_); a measure of barrier permeability to CuO NMs. For all experiments, CuSO_4_ was used as an ionic control.

**Results:**

CuO NMs and CuSO_4_ caused a concentration dependent decrease in cell viability in undifferentiated cells. CuO NMs and CuSO_4_ translocated across the differentiated Caco-2 cell monolayer. CuO NM mediated IL-8 production was over 2-fold higher in undifferentiated cells. A reduction in cell viability in differentiated cells was not responsible for the lower level of cytokine production observed. Both CuO NMs and CuSO_4_ decreased TEER values to a similar extent, and caused tight junction dysfunction (ZO-1 staining), suggesting that barrier integrity was disrupted.

**Conclusions:**

CuO NMs and CuSO_4_ stimulated IL-8 production by Caco-2 cells, decreased barrier integrity and thereby increased the P_app_ and translocation of Cu. There was no significant enhancement in potency of the CuO NMs compared to CuSO_4_. Differentiated Caco-2 cells were identified as a powerful model to assess the impacts of ingested NMs on the GI tract.

**Electronic supplementary material:**

The online version of this article (doi:10.1186/s12989-017-0211-7) contains supplementary material, which is available to authorized users.

## Background

Copper (Cu) is an essential micronutrient present in all tissues and is required for a plethora of cell functions including for example; peptide amidation, cellular respiration, pigment formation neurotransmitter biosynthesis and connective tissue strength [1, 2]. Cu has also been implicated in the development and maintenance of both innate and acquired immunity [3, 4]. The pathogenesis of many neurological diseases (e.g. Alzheimer’s disease, amyotrophic lateral sclerosis, Huntington’s disease, Parkinson’s disease) is associated with a disruption in Cu homeostasis [5, 6]. Excessive ingestion of copper by humans can cause gastrointestinal disturbance with symptoms such as nausea, vomiting, diarrhoea, and abdominal pain [7, 8].

Nanomaterials (NMs) have been used in wide ranging applications such as cosmetics, electronics, textiles, inks, pharmaceuticals and food contact materials [9, 10]. The anti- microbial properties of copper oxide nanomaterials (CuO NMs) are used in array of products such as textiles [11, 12], intrauterine devices [13], food contact materials [14] and wood preservation (due to its antifungal properties) [15]. Cu is relatively cheap and readily available and so the exploitation of CuO NMs has increased over recent years. For example, the antimicrobial properties of CuO NMs could promote its use as an alternative to silver and gold NMs in products, to reduce their manufacturing cost [16]. CuO NMs are also useful in heat transfer fluids and/or semiconductors [13, 17] and as inks [16, 18, 19].

A diverse array of NMs are available which vary with respect to their size, composition, surface area, charge, shape/structure and solubility. These physico-chemical properties can influence the biological response to NMs [20]. Metallic NMs (such as CuO) can be soluble, and thus may elicit toxicity via particle and/or ion mediated effects. For this reason, ionic (metal salt) controls are often included in hazard studies [21–23] and NM solubility is commonly assessed using ICP-MS. Compared to other engineered NMs (such as silver (Ag) and titanium dioxide (TiO_2_); there are a limited number of studies which have assessed the hazard potential of CuO NMs. Impacts on the lung in vivo [24, 25] and on lung cells in vitro have been investigated. For example it has been demonstrated that CuO NMs (42 nm) were the most potent in terms of cytotoxicity and DNA damage to the A459 human lung epithelial cell line, compared to zinc oxide (ZnO), iron complexes (CuZnFe_2_O_4_, Fe_3_O_4_, and Fe_2_O_3_) and TiO_2_ [26]. The toxicity of CuO NMs on the liver, kidney, spleen (assessed in vitro and in vivo) and zebrafish has also been assessed to a limited extent [27–32]. However, there are a lack of studies that have investigated the toxicity of ingested CuO NMs, with existing studies focusing on investigation of their antimicrobial properties (as exploitation of this NMs often relies on this property).

Ingestion of CuO NMs by humans is most likely to occur accidentally (e.g. due to leaching of CuO NMs from food contact materials into food). NMs may also enter the GI tract following inhalation in occupational, environmental and consumer settings due to clearance via the mucociliary escalator [33–35] or due to hand to mouth contact [36, 37]. Currently, little is known about the risks associated with NM ingestion, despite the potential increase in NM ingestion by humans [38, 39]. Addressing this knowledge gap is therefore a research priority.

Due to the large number of diverse NMs whose safety needs to be assessed, it is important to align nanotoxicology studies to the 3Rs principles (Replacement, Reduction and Refinement of animal testing) [40]. Accordingly, the toxicity of ingested CuO NMs was assessed in vitro in this study using the Caco-2 cell line, which originates from a human colon adenocarcinoma [41]. Culturing of Caco-2 cells for 15–21 days, leads to their spontaneous differentiation to mature enterocyte-like cells resembling the mature enterocytes of the small intestine in vivo, without growth factor supplementation [42–44]. During differentiation of the Caco-2 cell line, functional tight junctions joining the monolayers and well developed microvilli are formed at the apical (AP) membrane of mature enterocytes. [42]. In addition, the AP membrane of differentiated Caco-2 cell line expresses the characteristic hydrolases such as sucrose-isomaltase, lactase, aminopeptidase N and dipeptidyl peptidase IV characteristic of the absorptive enterocyte of small intestine microvilli [42, 45]. Undifferentiated Caco-2 cells have been most commonly used to assess the toxicity of NMs (e.g. TiO_2_, SiO_2_, ZnO, MgO, Ag) to the intestine in vitro [46–49]. The following endpoints have been prioritised within the assessment of NM toxicity to Caco-2 cells; cytotoxicity, cytokine production, oxidative stress and DNA damage as it is established that NMs often stimulate toxicity via inflammatory and oxidant driven responses. When differentiated Caco-2 cells have been used as a model to investigate the toxicity of ingested NMs, only limited endpoints (cytotoxicity, cytokine production, barrier integrity) have been assessed [50, 51]. Only one published study could be identified which investigated the toxicity of rod and spherical CuO NMs to differentiated Caco-2 cells [51]. It was found that rod shaped NMs were more toxic than the spherical shaped CuO NMs [51]. A comparison of the toxicity of SiO_2_ and ZnO NMs in undifferentiated and differentiated Caco-2 cells has been performed via assessment of cytotoxicity, cytokine production, and it was found that undifferentiated cells were more sensitive to NM toxicity compared to differentiated cells [50]. The use of undifferentiated cells is quicker and cheaper than using the differentiated Caco-2 model. However, differentiated cells more accurately mimic in vivo conditions. Therefore, comparisons in the sensitivity of undifferentiated and differentiated cells are useful when developing a testing strategy to assess the toxicity of ingested NMs.

Transepithelial electrical resistance (TEER) is commonly used to monitor Caco-2 cell differentiation during culture. TEER of differentiated Caco-2 cells ranges from 260 up to 1200 Ω.cm^2^ depending on the experimental design (e.g. medium composition in the AP and basolateral compartments, and cell seeding density) [52]. TEER measurement is also a parameter that is commonly used to ascertain the integrity and viability of the cell monolayer in toxicity studies [53]. To date, no studies could be identified which assessed the impact of NMs on the integrity of the Caco-2 intestinal barrier over time via measurement of TEER. Differentiated Caco-2 cells have been commomly used as an in vitro model to assess the translocation of substances (e.g. pharmaceuticals, pathogens) across the intestinal barrier [54, 55] and the rate of paracellular transport in differentiated Caco-2 cells has been demonstrated to be lower than that of the human intestine [56, 57].

Differentiated Caco-2 cells have been widely used to investigate the translocation of nanomedicines (e.g. polymers and liposomes) across the intestinal barrier, as reviewed Belogui et al. [58]. However, investigation of the translocation of other types of NMs has only been investigated to a limited extent previously. The translocation of CuO NMs across the Caco-2 intestinal barrier in vitro has only been investigated in one study. It was demonstrated that CuO NM transport was greater than that of CuSO_4_ [59], although the concentration of Cu was not standardized for CuSO_4_ and CuO NMs, making it challenging to compare the transport of these substances across the intestinal barrier. Of relevance is that the transport of other NMs has also been investigated in vitro and these studies have demonstrated how NM physico-chemical properties can influence their translocation. For example, translocation across the intestinal barrier and cytotoxic effects of a panel of zinc oxide (ZnO) NMs (of various sizes) has been studied using differentiated Caco-2 cells. It was observed that ZnO NMs (20 nm) had a higher level of permeation across the intestinal barrier and elicited greater cytotoxicity than larger particles (diameter of 1–5 μm or 90–200 nm) [60], demonstrating that particle translocation across the intestinal barrier may be a size dependent phenomenon. There is also evidence that gold NMs (1.4 to 200 nm) can translocate across the intestinal barrier of rats following ingestion [61]. However, in general there are a lack of studies which have investigated the influence of NMs on intestinal barrier integrity, and the impact this has on their translocation.

This study investigated the ability of CuO NMs and CuSO_4_ to stimulate cytotoxicity and cytokine (IL-8) production in differentiated and undifferentiated Caco-2 cells. The impact of CuO NMs and CuSO_4_ on barrier integrity (via assessment of TEER, tight junction staining and cell morphology (i.e. presence of microvilli)) and their transport across intestinal epithelial cells in vitro was assessed in differentiated Caco-2 cells. It is cheaper and quicker to perform studies using undifferentiated cells; however, the use of differentiated cells more accurately mimics the in vivo environment. Therefore, the advantages and limitations of using differentiated or undifferentiated Caco-2 models for assessment of NMs toxicity were also explored in this study.

## Methods

### Nanomaterial, characterisation and preparation

CuO NMs was obtained in powdered form from Plasma Chem, GmbH, Berlin, Germany, as a kind gift from project partners in the FP7 funded project Sustainable Nanotechnologies (SUN). The information data sheet from the supplier showed that it is a crystalline material with size of 15–20 nm, specific surface area of 47m^2^/g and a density of 6.3 g/cm^3^ as determined using the Brunauer–Emmett–Teller (BET) method. Detailed characterization of the size dissolution and surface chemistry of the CuO NMs by Transmission Electron Microscope (TEM), X-ray diffraction (XRD), and Inductive Coupled Plasma Optical Emission Spectrometry (ICP-OES) are available in Gosens et al. [25]. Briefly, CuO NMs has a primary particle size of 10 nm according to TEM analysis and 9.3 nm according to XRD. At pH 7.4 < 1.5% of CuO NMs were dissolved in Gamble’s solution at 1 and 24 h. In contrast at a pH of 4.5 approximately 62% of the CuO NMs had dissolved in Gamble’s solution at 1 and 24 h [25]. Copper sulphate (CuSO_4_) was purchased from Sigma (Poole UK).

CuO NMs and CuSO_4_ were dispersed following the procedure described by Jacobsen et al., [62]. Briefly, NMs or CuSO_4_ were dispersed in 2% FCS in Milli Q de-ionised water and sonicated continuously in a bath sonicator for 16 min. CuO NMs or CuSO_4_ were then immediately diluted in cell culture medium (see below) to the required concentrations. The hydrodynamic diameter, zeta potential and polydispersity index (PdI) of CuO NMs in biological medium were determined using Dynamic Light Scattering (DLS, Malvern Zeta sizer Nano series) at 0 h and at 24 h (following incubation at 37 °C). Following dispersion by sonication in 2% FCS, the concentration was adjusted to 50 μg/ml Cu in phenol red free cell culture medium and the hydrodynamic diameter, PdI and Zeta potential were measured.

### Cell culture

The human colon colorectal adenocarcinoma (Caco-2) cell line was obtained from the American Type Culture Collection (ATCC) (USA). The cells were maintained in minimum essential medium eagle (MEM) (Sigma) supplemented with 10% heat inactivated fetal bovine serum (FBS) (Gibco Life Technologies), 100 U/ml Penicillin/Streptomycin (Gibco Life Technologies), 100 IU/ml nonessential amino acid (NEAA) (Gibco Life Technologies, Paisley, UK), and 2 mM L- glutamine (L-Glu) (Gibco Life Technologies) (termed complete cell culture medium) at 37 °C and 5% CO_2_. The cells were sub-cultured using trypsin-EDTA (Gibco Life Technologies).

### Alamar blue cell viability assay: undifferentiated cells

Caco-2 cells were seeded at a concentration of 1.56 × 10^5^ cells/cm^2^ into the wells of a 96 well plate (surface area 0.32 cm^2^) (Coaster Corning Flintshire, UK) and incubated for 24 h at 37 °C and 5% CO_2._ At this time 100% confluency was reached. The cell culture medium was then removed, the cells were washed twice with phosphate buffered saline (PBS) (Gibco Life Technologies) and exposed to 100 μl of either cell culture medium (control), 0.1% Triton-X 100 (positive control) or Cu concentrations. The concentration of Cu in CuO NMs and CuSO_4_ was standardised to ensure that cells were exposed to an equivalent concentration of Cu for each treatment, in all experiments. These concentrations were expressed on a mass basis of micro gram of Cu per centimetre square (Cu μg/cm^2^). The concentrations used were 0.37 to 78.13 μg/cm^2^ Cu, which is equivalent to 1.95 to 250 μg/ml.

At 24 h post-exposure, the cell supernatant was removed, stored at -80 °C and replaced with alamar blue reagent (100 μl, Sigma, Poole, UK) diluted to 0.1 mg/ml in cell culture medium. The cells were incubated for 4 h at 37 °C, 5% CO_2_ and fluorescence measured at 560/590 nm (excitation/emission). Data was analysed using PROAST 38.9 software to obtain the Benchmark dose-response (BMD) 20 (the concentration of CuO NMs that increase cell death by 20%) and cell viability expressed in percentage of the untreated control. Concentrations of 2.22, 3.17, 4.44, 6.34, 8.88, 12.68 μg/cm^2^ were selected for further study based on the findings from the Alamar blue assay.

### Caco-2 cell differentiation

To obtain a differentiated Caco-2 cell monolayer, cells were seeded at a concentration of 3.13 × 10^5^ cells/cm^2^ in 500 μl cell culture medium which were added into the apical compartment of 3.0 μm pore polycarbonate transwell inserts of a 12- well plate with growth area of 1.12 cm^2^ (Corning, Flintshire, UK). The basolateral (lower) compartments were filled with 1.5 ml of cell culture medium. The cells were cultured at 37 °C, 5% CO_2_ and 95% humidity for 16–21 days. The medium was changed every other day for the first 14 days and then every day until day 21 with fresh medium.

### Measurement of trans-epithelial electrical resistance (TEER)

Trans-epithelial electrical resistance (TEER) were measured using an epithelial volt-ohmmeter EVOM2 (World precision instrument, Sarasota, USA). The resistance reading (in ohms) was taken once the reading had stabilized and measurements were taken every 2 days until day 21. The resistivity was calculated using Eq. 1.

1 $$ Resistivity\left(\varOmega .{cm}^2\right)= ohm2- ohm1\times A $$

Where ohm1 = Resistance of the insert with cell culture medium only.

ohm 2 = Resistance of the insert with cell.

A = surface area of the insert in cm^2^.

TEER are reported as resistivity.

Only Caco-2 cell monolayers with TEER values greater than 500 Ω.cm^2^ were used for experiments. The impact of CuO NMs and CuSO_4_ on the barrier integrity of differentiated Caco-2 cells was studied by measuring TEER. Differentiated cells were exposed to cell culture medium (negative control), 0.1% triton X100 (positive control), CuO NMs or CuSO_4_ (6.34 and 12.68 μg/cm^2^) and TEER measurements taken every 3 h for 15 h and at 24 h post exposure starting from time 0 (immediately after treatment with CuO NMs and CuSO_4_).

### Immunostaining of differentiated Caco-2 tight junctions

Differentiated Caco-2 cells were exposed to cell culture medium (control), CuO NMs or CuSO_4_ (6.34 μg/cm^2^) and washed twice with PBS. The cells were fixed with 4% formaldehyde for 25 min at RT and excess aldehyde groups were quenched with 50 mM ammonium chloride for 10 min at RT. The cells were permeabilized with 0.1% triton X100 for 10 min and blocked for nonspecific binding with 10% BSA for 2 h at RT. Cells were then incubated with 1 μg/ml anti-ZO1 tight junction protein antibody (Abcam, Cambridge, UK) diluted in 1% BSA overnight (o/n) at 4 °C. Next, cells were incubated with a secondary antibody, Alexa Fluor 488 goat anti-rabbit IgG H&L (Abcam, Cambridge, UK) diluted to 4 μg/ml with 1% BSA for 1 h followed by nuclear staining with 4, 6- diamido-2-phenylindole (DAPI, 300 nM) for 15 min at RT. Staining with DAPI was used to visualise the nuclei of cells and to assess cell number (as an indicator of cell viability). Cells were washed three times with PBS after each step. The polycarbonate inserts on which the differentiated Caco-2 cell monolayer was grown were carefully excised, mounted with mowoil and covered with a glass coverslip, which was sealed with nail polish. Cells were visualized using Zeiss LSM880 confocal microscope for ZO-1 or Zeiss fluorescent Microscope, Carl Zeiss Axio Scope A 1 Upright Research Microscope for nuclear counting (Germany) and the results analysed using the Zen program and Image J software. Three fields of view (each of which was 140.80 X 105.60 μm) were analysed for each sample and the results were presented in mean number cells ± SEM and then representative images presented.

### Imaging of cell morphology using light microscopy

Differentiated Caco-2 cells (3.13 × 10^5^ cells/cm^2^) were grown for 21 days on 3.0 μm pore polycarbonate transwell inserts of a 12- well plate (Coaster Corning, Flintshire, UK) while undifferentiated Caco-2 cells (3.13 × 10^5^ cells/cm^2^) were grown on a glass coverslip in a 24 well plate (Coaster Corning, Flintshire, UK) for 24 h. Both the differentiated and undifferentiated Caco-2 cells were exposed to cell culture medium (control), 6.34 μg/cm^2^ Cu of CuO NMs or CuSO_4_. After 24 h, the cells were stained with Rapid Romanowsky stain (TCS Biosciences, England). Rapid Romanowsky stain combines the basic (cationic) dye, methylene blue (azure) and the acid (anionic) dye, eosin Y. The basic dye binds acid nuclei thereby generating a purple colour while the acid dye binds to the cytoplasm producing a red colour [63]. Briefly, Caco-2 cells were fixed with methanol, stained with eosin Y and counter stained with methylene blue. The cells were washed in distilled water dried and mounted with DPX mountant for histology (Sigma, Poole UK). The cell were examined using light microscopy (magnification 40X).

### Scanning electron microscopy (SEM)

For differentiated Caco-2 cells, 3.13 × 10^5^ cells/cm^2^ were grown for 21 days on 3.0 μm pore polycarbonate transwell inserts of a 12- well plate, with growth area of 1.12 cm^2^ (Coaster Corning, Flintshire, UK). For undifferentiated Caco-2 cells, 3.13 × 10^5^ cells/cm^2^ were grown on a glass coverslip in a 24 well plate (Coaster Corning, Flintshire, UK) for 24 h. Both the differentiated and undifferentiated Caco-2 cells were treated with 12.68 μg/cm^2^ Cu of CuO NMs. After 24 h, the cells were washed with PBS twice, fixed with 5% glutaraldehyde in 0.1 M sodium cacodylate for 2 h at 4 °C. The cells were washed thrice with 0.1 M sodium cacodylate and dehydrated in graded ethanol (25, 50, 70, 80 and 90%) for 10 min in each ethanol grade at room temperature. The cells were further dehydrated in 100% ethanol thrice for 15 min and then submerged in 2:1 fresh solution of hexamethyldisilazane (Sigma):100% ethanol. The cells were finally dried in 100% hexamethyldisilazane (Sigma), coated with gold and examined with Focus Ion Beam Scanning Electron Microscopy (FIB/SEM).

### Cytokine analysis

Differentiated Caco-2 cells were exposed to cell culture medium (negative control), 200 ng/ml tumour necrosis alpha (TNF-α) (positive control) 3.17, 6.34, or 12.68 μg/cm^2^ Cu of CuO NMs and CuSO_4_ for 24 or 48 h. Undifferentiated Caco-2 cells were grown in a 96 well plate with a surface area of 0.32 cm^2^ (Coaster Corning Flintshire, UK). A concentration of 1.56 × 10^5^ cells/cm^2^ was seeded into the wells of the plate and incubated for 24 h at 37 °C and 5% CO_2_ until 100% confluency was reached. Cells were then exposed to cell culture medium (control), 200 ng/ml TNF-α (positive control), 2.22, 3.17, 4.44, 6.34, 8.88 and 12.68 μg/cm^2^ Cu of CuO NMs or CuSO_4_ for 6 or 24 h. The cell supernatants were collected from undifferentiated cells, and the apical and basolateral compartments of differentiated cells and stored in −80 °C until required. On the day of cytokine analysis, the supernatants were thawed and IL-8 levels quantified using Enzyme-linked Immunosorbent assay (ELISA). Human IL-8 duo set ELISA kits were purchased from R&D System, Inc., (Minneapolis, MN USA) and used for the cytokine analysis according to the manufacturer’s protocol. Human IL-8 production was measured using a microplate reader, SpectraMax M5 (California USA) at wavelength 450 nm.

### Translocation studies

Differentiated Caco-2 cells were treated with cell culture medium (control), 3.17, 6.34 and 12.48 μg/cm^2^ Cu of CuO NMs or CuSO_4_ for 24 or 48 h at 37 °C, 5% CO_2_and 95% humidity and the cell culture medium then were removed from apical and basolateral compartments. Apical (300 μl) and basolateral (900 μl) media was digested with 5 ml of 4% HNO_3_ (Sigma), filtered with Puradisc 25 mm 0.2 μm PES filter media (Whatman) and made up to 10 ml with Milli Q deionised H_2_O to obtain final acidic concentration of 2% HNO_3_. For cell preparation, the cells were digested following the method described by Bolea et al. [64]. Briefly, the cells were detached using 100 μl of 25 mM trypsin EDTA and 20% HNO_3_ (1 ml) was added to the cells. The cells were then shaken with a rotatory shaker, PMS-1000, Grant-bio (Cambridge UK) at high speed for 4 h, at RT. The solution was then diluted with Milli Q H_2_O to get an acidic concentration of 2% HNO_3_, and then filtered with Puradisc 25 mm 0.2 μm PES filter media (Whatman). The acidic extracts of medium and cell were analysed by ICP-OES using a Perkin Elmer Optima 5300 DV (USA), employing an RF forward power of 1400 W, with argon gas flows of 15, 0.2 and 0.75 L/min for plasma, auxiliary, and nebuliser flows, respectively.

Apparent permeability coefficients (P_app_) of Cu was calculated using Eq. 2 [65].

2 $$ Papp\left( cm/s\right)=\frac{\Delta Q}{\Delta t}\times \frac{1}{A}\times Co $$

Where ∆Q/∆t is the amount of Cu transported into the basolateral compartment per unit time (t), A is the surface area of the insert (Caco-2 cell monolayer) and Co is the initial concentration of the substance in the donor (apical) compartment.

### Data analysis

Each experiment was repeated at least three times (on different days) for undifferentiated and differentiated cells and all data generated from these experiments are expressed as the mean ± standard error of the mean (SEM). The figures were generated using Graph pad Prism. After checking normality of the data, a one-way analysis of variance (ANOVA) followed by the Tukeys multiple comparison was employed to investigate statistical significance using Minitab 17 software. PROAST version 38.9 software was used to analyse Benchmark dose-response.

## Results

### Physico-chemical characteristics of the CuO NMs

Here, we analysed the hydrodynamic diameter, zeta potential and polydispersity index (PdI) of CuO NMs in cell culture medium at 0 and 24 h post incubation at 37 °C 5%, CO_2_ and 95% humidity. CuO NMs were highly agglomerated in complete cell culture medium at 0 h, as the average hydrodynamic diameter was 157 nm, whilst the primary particle size measured by TEM was 10 nm [25]. After incubation for 24 h at 37 °C, 5%, CO_2_ and 95% humidity in complete cell culture medium, a significant decrease in hydrodynamic diameter (to 24 nm) and PdI was observed. The zeta potential remained constant and negative at 0 and 24 h. The PdI at 0 and 24 h time points were less than one, indicating that CuO NMs suspensions were suitable for DLS analysis (Table 1).

**Table 1:** Hydrodynamic diameter, zeta potential and PdI values after dispersion of 50 μg/ml Cu of CuO NMs in complete cell culture medium

Time (h)	Hydrodynamic diameter (nm)	Zeta Potential	PdI
0	157.37 ± 29.44*	−7.38 ± 0.59	0.6 ± 0.07*
24	24.01 ± 0.56	−7.29 ± 0.05	0.42 ± 0.1

CuO NMs were dispersed in 2% FCS in water, sonicated and then diluted in complete cell culture medium and the hydrodynamic diameter, zeta potential and PdI were measured immediately (0 h) or following incubation at 37 °C, 5%, CO_2_ and 95% humidity for 24 h. Data expressed as Mean ± SEM (*n* = 3). Asterisk (*) represents significance when incubation at time point 0 and 24 h are compared *P* < 0.05

The solubility of CuO NMs following dispersion in MEM and DMEM was analysed using ICP-OES and showed that 47.79 and 53.53% CuO NMs dissociated to Cu^2+^ in MEM after 1 and 24 h respectively and in DMEM this was 59.91 and 67.41% at 1 and 24 h (Additional file 1). This indicates greater solubility of CuO NMs in medium compare to Gamble’s solution at physiological pH, which was previously identified to be ~1.5% [25]. Further information on CuO NMs characterisation from existing studies is provided in the methods section.

### Impact of CuO NMs on viability of undifferentiated Caco-2 cell

**Fig. 1:**
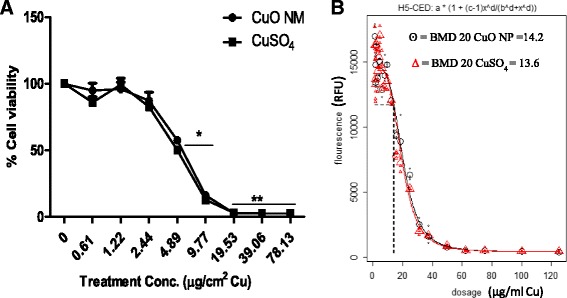
Cytotoxicity of CuO NMs and CuSO_4_ to undifferentiated Caco-2 cells. Viability of undifferentiated Caco-2 cells was assessed using the alamar blue assay following exposure of cells to cell culture medium (control), CuO NMs or CuSO_4_ at concentrations ranging from 0.61 and 78.13 μg/cm^2^ Cu for 24 h. **a**) Viability of Caco-2 cells following CuO NMs or CuSO_4_ exposure expressed as a % of the control. **b**) Determination of 20% Benchmark dose (BMD 20) in μg/ml following exposure of undifferentiated Caco-2 cells to CuO NMs or CuSO_4_ exposure. Data was analysed using Proast 38.9 software to obtain the BMD 20. Data are expressed in mean ± SEM (*n* = 3) and * represents significance compared to control at *P* < 0.05

A concentration dependent decrease in undifferentiated Caco-2 cell viability was observed after treatment with CuO NMs and CuSO_4_ for 24 h (Fig. 1a). At concentrations below 1.22 μg/cm^2^Cu of CuO NMs, there was little or no impact on the viability of undifferentiated Caco-2 cells. Cell viability significantly decreased in a concentration dependent manner on exposure to 2.44 to 19.53 μg/cm^2^ Cu of CuO NMs reaching 90% cell death at concentrations above 19.53 μg/cm^2^ Cu of CuO NMs after 24 h. CuSO_4_ also followed the same pattern. No significant difference between the toxicity of CuO NMs and CuSO_4_ was observed when concentration was expressed as μg/cm^2^ Cu (Fig. 1a). Using Proast 38.9 software, the concentration of CuO NMs and CuSO_4_ required to kill 20% of the cells were 4.44 and 4.25 μg/cm^2^ Cu respectively (Fig. 1b). This informed the selection of the following sub-lethal concentrations of CuO NMs and CuSO_4_ (2.22, 3.17, 4.44, 6.34, 8.88 and 12.68 μg/cm^2^ Cu) to test in further studies.

### Impact of CuO NMs and CuSO_4_ on morphology and integrity of differentiated Caco-2 cell monolayer

**Fig. 2:**
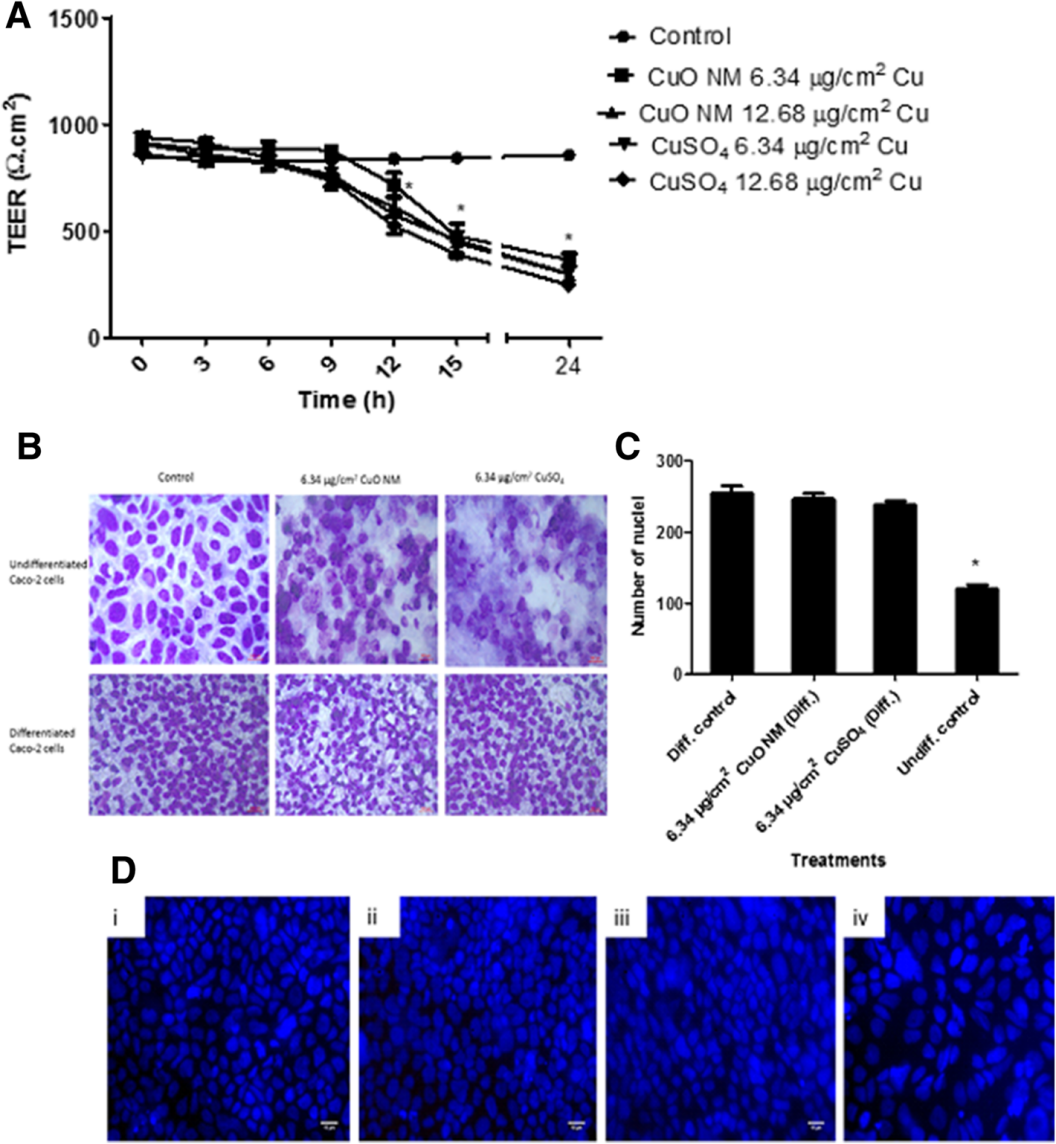
Impact of CuO NMs and CuSO_4_ on the differentiated and undifferentiated Caco-2 cell monolayer. **a**) Impact of CuO NMs and CuSO_4_ on differentiated Caco-2 cell TEER values. Following differentiation, Caco-2 cells were exposed to cell culture medium (control, 0), CuO NMs or CuSO_4_ at concentrations of 6.34 or 12.68 μg/cm^2^ Cu at the apical compartment. The TEER values were measured using epithelial volt-ohmmeter EVOM every 3 h. Data are expressed in mean TEER value ± SEM (*n* = 3) and * represents significant difference compared to control at *P* < 0.05. **b**) Differentiated and undifferentiated Caco-2 cell morphology following exposure to CuO NM or CuSO_4_. Cells were exposed to control (cell culture medium) and 6.34 μg/cm^2^ Cu CuO NM or CuSO_4_ for 24 h. The cells were fixed, stained and visualised using the light microscopy (magnification 40X, scale bar = 500 μm. **c**) Total nuclei count of differentiated and undifferentiated Caco-2 cells. **d**) Representative image of nuclei staining with DAPI (field of view: 140.80 X 105.60 μm). i) Untreated differentiated Caco-2 cell, ii) differentiated Caco-2 cell treated with 6.34 μg/cm^2^Cu CuO NM, iii) differentiated Caco-2 cell treated with 6.34 μg/cm^2^Cu CuSO_4_ and iv) untreated undifferentiated Caco-2 cell. For **c** and **d**, the nucleus were stained with DAPI and the images obtained with Zeiss fluorescent Microscope, Carl Zeiss Axio Scope A 1 Upright Research Microscope (magnification 40X) and the results analysed using Image J software. Scale bar = 10 μm

**Fig. 3:**
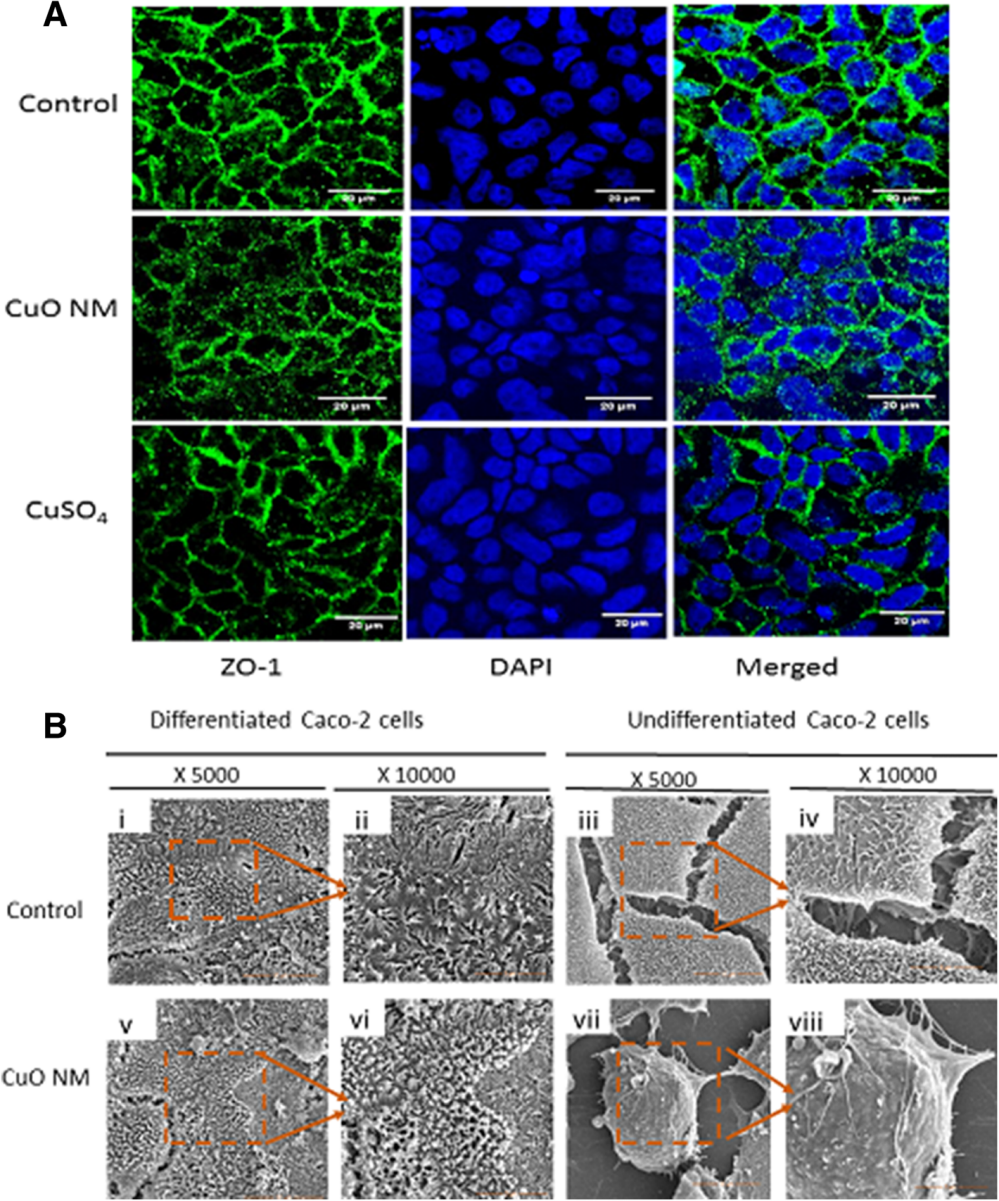
The impact of CuO NMs and CuSO_4_ on Caco-2 cell morphology. **a**) Following differentiation, cell monolayers were exposed to cell culture medium (control) or 6.34 μg/cm^2^ Cu of CuO NMs and CuSO_4_ for 24 h and then fixed and stained for the tight junction protein ZO-1 (green) and nucleus with DAPI (blue). The images were obtained with Zeiss LSM880 confocal microscope using the Zen program for data analyses. Scale bar = 20 μm. **b**) Assessment of cell morphology using SEM. Differentiated and undifferentiated Caco-2 cells were exposed to 12.68 μg/cm^2^ Cu of CuO NMs for 24 h and then were washed, fixed, dehydrated, dried and examined by FIB/SEM. i and ii are control differentiated Caco-2 cells enlarged X 5000 and X 10000 respectively. iii and iv are control undifferentiated Caco-2 cells enlarged X 5000 and X 10000 respectively. v and vi are differentiated Caco-2 cells exposed to 12.68 μg/cm^2^ Cu of CuO NMs enlarged X 5000 and X 10000 respectively and vii and viii are undifferentiated Caco-2 cells exposed to 12.68 μg/cm^2^ Cu of CuO NMs enlarged X 5000 and X 10000 respectively

Differentiation of Caco-2 cell monolayer was evidenced by the maintenance of TEER value between 823.67 and 861.33 Ω.cm^2^ over 21 days, which showed no significant difference over the duration of the experiment (24 h) for control cells (Fig. 2a). Staining for the tight junction protein Zonula occludens −1 (ZO-1) (Fig. 3a) also confirmed that the differentiation of Caco-2 was successful. In contrast, no tight junction protein (Additional file 2) or microvilli were observed for undifferentiated Caco-2 cells. According to SEM images (Fig. 3b), both differentiated and undifferentiated Caco-2 control cells covered the entire surface, indicating that they were confluent. However, differentiated Caco-2 control cells had microvilli, which were lacking in undifferentiated Caco-2 cells.

The TEER value of the differentiated Caco-2 cell monolayer treated with 6.34 and 12.68 μg/cm^2^ Cu of CuO NMs and CuSO_4_ respectively remained relatively constant and equivalent to the control until 9 h post exposure. At 12 and 15 h, CuO NMs and CuSO_4_ induced a significant decrease in TEER values at all concentrations tested (Fig. 2a). At 24 h, the greatest reduction in TEER value was observed; the TEER value for both CuO NMs and CuSO_4_ was significantly lower than negative control cells at both concentrations tested (6.34 and 12.68 μg/cm^2^ Cu). For the positive control (Triton X100), the TEER value decreased to 23 ± 0.52 Ω.cm^2^ after 3 h. There was no significant difference between the changes in TEER induced by either CuO NMs or CuSO_4_ at all-time points investigated. Cells treated with 6.34 μg/cm^2^ Cu of CuO NMs or CuSO_4_ for 24 h also exhibited a reduced degree of tight junction protein staining compared to control (Fig. 3a).

The microvilli of differentiated Caco-2 cells exposed to CuO NMs were shortened and crypt-like (Fig. 3b). However, there was no obvious loss of cells. It was not possible to perform the Alarmar Blue assay on the transwell insert used to culture differentiated cells, or conduct the LDH assay due to Cu interference in this assay [66]. Therefore, in order to assess the impact of CuO NMs and CuSO_4_ on the viability of differentiated cells, cells were imaged using light microscopy, and in addition, cell number was counted via DAPI staining. Light microscopy (Fig. 2b) and SEM (Fig. 3b) images revealed that there was a loss of undifferentiated Caco-2 cells following exposure to CuO NMs or CuSO_4_ however, no loss of differentiated cells was observed. There were significantly less undifferentiated control cells, as indicated by a decrease in nuclei number, compared to differentiated Caco-2 cells (in an equivalent field of view: 140.80 X 105.60 μm) (Fig. 2c and d). No change in the number of differentiated cells was observed following exposure to CuO NMs or CuSO_4_ for 24 h (Fig. 2c and d). These findings suggest that CuO NMs or CuSO_4_ do not affect viability of differentiated Caco-2 cells at the concentrations and time point tested.

### IL-8 production by differentiated and undifferentiated Caco-2 cells

**Fig. 4:**
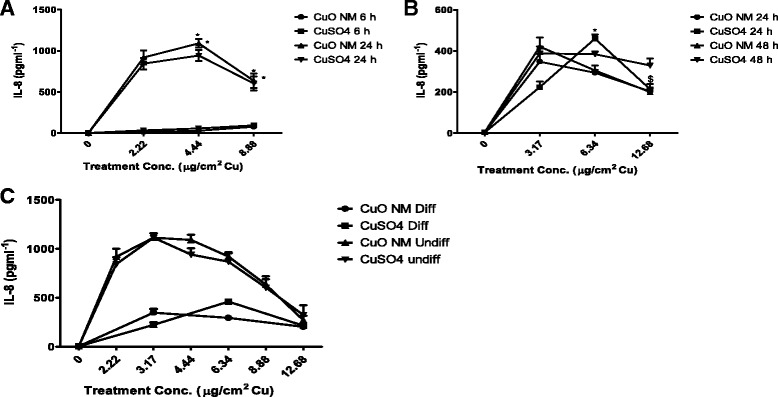
IL-8 production by differentiated and undifferentiated Caco-2 cells following CuO NMs and CuSO_4_ exposure. Cells were exposed to cell culture medium (control, 0), CuO NMs or CuSO_4_ at concentrations of < 12.68 μg/cm^2^ Cu for 6 or 24 h for undifferentiated cells and 24 or 48 h for differentiated cells. The level of IL-8 in the cell supernatant was determined using an ELISA kit. **a**) Comparison of 6 and 24 h time point of undifferentiated Caco-2 cells. **b**) Comparison of 24 and 48 h time point of differentiated Caco-2 cells. **c**) Comparison of differentiated and undifferentiated Caco-2 cells. Data are expressed as average IL-8 concentration (pg/ml) ± SEM (*n* = 3). Significance at *p* < 0.05 is indicated by * for comparison of different treatment concentrations or $ for comparison of 24 and 48 h time point

At 6 h undifferentiated cells exposed to CuO NMs or CuSO_4_ did not stimulate a significant increase in IL-8 production compared to control (Fig. 4a). In contrast, at 24 h undifferentiated Caco-2 cells treated with CuO NMs and CuSO_4_ demonstrated a significant increase in IL-8 production with up to approximately 1000 pg/ml of IL-8 produced at a concentration of 4.44 μg/cm^2^. For differentiated cells, IL-8 production increased to approximately 400pg/ml following exposure to both CuO NMs and CuSO_4_ at 24 and 48 h post exposure, compared to control (Fig. 4b). Both CuO NMs and CuSO_4_ therefore stimulated a concentration and time dependent increase in IL-8 production in both differentiated and undifferentiated Caco-2 cells. However, comparison of IL-8 production by differentiated and undifferentiated Caco-2 cells demonstrated that the cytokine production at 24 h was significantly greater for undifferentiated Caco-2 cells (Fig. 4c). The lower level of cytokine production observed in differentiated cells is unlikely to be a consequence of a loss of cell viability (Fig. 2b and c). There was no significant difference in IL-8 production between CuO NMs and CuSO_4_ treated cells for either differentiated or undifferentiated Caco-2 cell models. Differentiated and undifferentiated Caco-2 cells treated with 200 ng/ml TNF-α as positive control secreted 89.60 ± 4.77 and 275 ± 35.04 pg/ml respectively. IL-8 was below the limit of detection in the medium collected at the basolateral compartment of differentiated Caco-2 cells (data not shown).

### Translocation of copper across differentiated Caco-2 monolayer

**Fig. 5:**
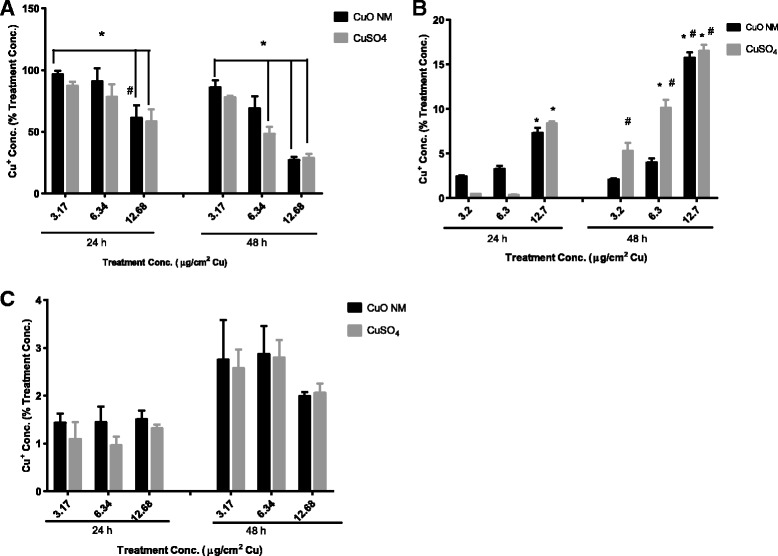
Cellular uptake and translocation across differentiated Caco-2 cells. Following differentiation of Caco-2 cells, cells were exposed to cell culture medium (control, 0), CuO NMs or CuSO_4_ at concentration of 3.17, 6.34 or 12.68 μg/cm^2^ Cu at the apical compartment for 24 and 48 h. The level of Cu in the apical, basolateral compartment and the cell were determined by ICP-OES. **a**) Apical compartment. **b**) Basolateral compartment, **c**) Cell. Data are expressed as mean copper concentration (as a percentage of the treatment concentration) ± SEM (*n* = 3). Significance *p* < 0.05 are indicted by * for comparison of treatment concentration or # for comparison of 24 and 48 h time point

In all experiments assessing the translocation of Cu across the differentiated Caco-2 cell monolayer, CuO NMs or CuSO_4_ were added directly to the apical compartment at time 0 h. The concentration of soluble Cu in the apical chamber of the cells was both concentration and time dependent. For the lowest concentration of CuO NMs and CuSO_4_ almost the entire initial dose (87.26 ± 3.88 to 96.35 ± 3.15%) was detectable in the apical chamber after 24 h of exposure, declining slightly (77.91 ± 1.01 to 85.74 ± 5.83%), after 48 h. In contrast, for the highest concentration of CuO NMs and CuSO_4_ approximately 58.07 ± 4.29 to 61.17 ± 10.10% was detectable in the apical compartment after 24 h exposure with a significant (*P* < 0.05) further decrease to between 27.00 ± 2.70 to 28.77 ± 3.30% after 48 h exposure (Fig. 5a).

Again, translocation measured as the content of copper in the basolateral chamber was concentration and time dependent. Therefore, the concentration of Cu in the basolateral media was greatest for cells exposed to CuO NMs and CuSO_4_ at the highest concentration of 12.68 μg/cm^2^ Cu, and longest time point of 48 h. Transport of Cu to the basolateral compartment also increased significantly from 24 h to 48 h for CuSO_4_ (3.17 and 6.34 μg/cm^2^ Cu respectively) at 24 h post exposure of the same concentration (Fig. 5b). The cellular uptake of CuO NMs or CuSO_4_ was <3% of the initial exposure dose at 24 and 48 h, for all concentrations tested (Fig. 5c). There was no significant retention of Cu in the cell monolayer for both CuO NMs and CuSO_4_ and Cu was not detected in the untreated control cells. No difference was observed between CuO NMs and CuSO_4_ for transport and uptake.

**Fig. 6:**
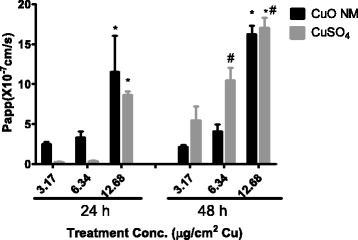
Apparent permeability coefficient (P_app_) of CuO NMs and CuSO_4_ across Caco-2 cells. Following differentiation Caco-2 cells were exposed to cell culture medium (control, 0), CuO NMs or CuSO_4_ at concentration of 3.17, 6.34 or 12.68 μg/cm^2^ Cu at the apical compartment for 24 and 48 h. The concentration of Cu in the apical and basolateral compartment were determined by ICP-OES. P_app_ was calculated using Eq. 2 (refer to Methods). Data are expressed in mean P_app_ ± SEM ×10^−7^ cm/s (*n* = 3). Significance *p* < 0.05 are indicted by * for comparison of treatment concentration or # for comparison of 24 and 48 h time point

When the data is re-expressed as the apparent permeability coefficient (P_app_), again a time and concentration dependent increase in permeability was observed following exposure of differentiated Caco-2 cells to CuO NMs and CuSO_4_. Both CuO NMs and CuSO_4_ significantly increased P_app_ of the Caco-2 cell monolayer at 24 and 48 h at a concentration of 12.68 μg/cm^2^ Cu. At 48 h CuSO_4_ stimulated a greater enhancement in P_app_ than CuO NMs, with a significant increase in permeability observed at concentrations of 14.2 and 28.4 μg/ml Cu (Fig. 6).

## Discussion

Despite the anticipated increase in CuO NM ingestion by humans associated with their increased use, there is a lack of understanding about the toxicity of CuO NMs to the GI tract. Indeed, assessment of the hazard of ingested NMs is recognised as a research priority as only limited number of studies have assessed the toxicity of ingested NMs [38, 67]. This study clearly demonstrates that the impacts of CuO NMs are entirely comparable to CuSO_4_ in a standard differentiated in vitro Caco-2 model of the GI tract for endpoints spanning TEER, cell morphology, tight junction integrity, translocation and IL-8 production. The comparable results of CuSO_4_ suggest that CuO NMs induced its effect, in part by an ion mediated mechanism.

### Cytotoxicity

Viability studies were carried out using undifferentiated Caco-2 cells to obtain a BMD 20 value. BMD is useful in estimating the concentration of a toxicant required to elicit a low, but measurable (sub lethal), toxic response. BMD eliminates the problems associated with determination of the of no-observed-effect-level (NOEL) as it uses full dose-response data in the statistical analysis thereby enhancing quantification of uncertainty in the data [68]. Although differentiated Caco-2 cells are considered to be more representative of cells in vivo, undifferentiated cells were used initially to screen cytotoxicity, as it is technically easier, quicker and cheaper to perform these studies and so a wider range of concentrations can be tested. Furthermore, the transwell inserts used to support the growth of differentiated Caco-2 cells are not compatible with the Alamar Blue Assay (which was used to assess the viability of undifferentiated cells). CuO NMs are also known to interfere with LDH assay (which uses the cell supernatant to assess cytotoxicity) [66] making it unreliable to perform this assay for CuO NMs. Therefore, it is more challenging to evaluate cytotoxicity and derive BMD with differentiated Caco-2 cells in transwell inserts. Accordingly, the viability of differentiated Caco-2 cells was assessed by visualising cell morphology using light microscopy, and staining cells with DAPI to count cell number.

CuO NMs stimulated a concentration dependent decrease in viability in undifferentiated cells. No significant difference was observed between the cytotoxicity of CuO NMs and CuSO_4_. Whilst CuO NMs and CuSO_4_ exhibit a similar level of toxicity, the CuO NMs tested in this study are not 100% soluble at 24 h, and thus it is likely that the CuO NMs exhibit toxicity via particle and ion effects. Previous studies which has demonstrated that Cu ions are likely to contribute predominantly to the toxicity of CuO NMs in differentiated Caco-2 cells [51] although they reported that particle shape could also contribute to the toxicity of CuO NMs. Furthermore, whilst the CuO NMs tested in our study exhibited some solubility in the cell culture medium, it is also possible that some ion release occurs inside the cell following particle uptake (e.g. Trojan horse mechanism), contributing further to Cu mediated effects. In comparison with the findings of other published studies using Caco-2 cells [50, 69], it seems that this study has demonstrated higher toxicity of CuO NMs than previously reported for ZnO (10 nm), SiO_2_ (14 nm), TiO_2_, iron oxides and Ag (20 nm) NMs although this would require verification in side-by-side tests. Data from cytotoxicity testing was used to inform the selection of CuO NM and CuSO_4_ concentrations to test when assessing impacts on differentiated cells. Of relevance is that imaging of cell morphology using light microscopy, and SEM revealed that there was a loss of cells in undifferentiated Caco-2 cells exposed to CuO NMs, which suggests that there was a reduction in cell viability. In contrast, no loss of differentiated cells was observed, which suggests that the concentrations and time point tested with CuO NMs were less toxic to the differentiated cells.

### Impact on barrier integrity

We used measurement of TEER in combination with assessment of tight junction protein (ZO-1) staining and visualisation of cell morphology (SEM e.g. presence of microvilli) to confirm the differentiation status of Caco-2 cells in our study. It is noteworthy that many other studies have only measured TEER values to assess the differentiation status of cells [51, 70], as this is an accepted method of confirming Caco-2 cell differentiation. However, in this study the use of three approaches (TEER measurement, tight junction staining and assessment of microvilli formation) provided a robust assessment not only of the differentiation status of the Caco-2 cells but also enabled the impact of CuO NMs on the intestinal barrier to be investigated.

Assessment of TEER over time has not been used as a measure of toxicity in previous studies which have investigated the impact of NMs on differentiated Caco-2 cells. The TEER values of differentiated Caco-2 cells showed a significant decrease from 9 h post-treatment with both CuO NMs and CuSO_4_ indicating a disruption in barrier integrity. To further study the impact of CuO NMs and CuSO_4_ the tight junction protein (ZO-1) of differentiated Caco-2 cells was stained and imaged. A decrease in tight junction protein staining was observed in treated cells, which confirms that CuO NMs and CuSO_4_ compromised the integrity of the Caco-2 monolayer. The images of ZO-1 staining after treatment with CuO NMs and CuSO_4_ are less clear, but this is due to disruption of the tight junctions (confirmed by TEER and SEM) rather than poor staining or imaging. In addition, SEM imaging confirm the barrier perturbation and/or toxicity of CuO NMs and CuSO_4_ on differentiated Caco-2 cell monolayer integrity. Impairment of barrier integrity has a number of implications for the function of the intestine. For example, the permeability of the intestinal barrier is likely to increase due to tight junction dysfunction, potentially permitting the transport of substances (e.g. chemicals, pathogens) across the intestinal barrier.

Translocation of NMs (polystyrene) across the intestinal barrier has been observed in vivo [71], however NM penetration (excluding nanocarriers) across the intestinal barrier has not been widely studied in vitro, despite the extensive use of the differentiated Caco-2 model to study the translocation of pharmaceuticals and pathogens [54, 55]. Translocation across the intestinal barrier determines the bioavailability of NMs after oral exposure and could be affected by NMs physico-chemical properties including size, charge, time and the experimental set up (e.g. concentration, time point) [72, 73]. In this study, it was demonstrated that CuO NMs and CuSO_4_ translocated across the intestinal barrier in a concentration and time dependent manner. The transport of Cu is likely to be facilitated by the ability of CuO NMs and CuSO_4_ to disrupt barrier integrity. More specifically, a loss of tight junction function (as indicated by measurement of TEER value and tight junction protein staining in this study) is likely to enhance paracellular NM translocation. In vitro studies have demonstrated up to 7.8% and 0.8% translocation of 50 and 100 nm charged polystyrene NMs respectively using Caco-2 co-culture intestinal epithelial cell models [72] which is similar to the findings of this study.

At the lowest concentration of CuO NMs and CuSO_4_ tested summation of copper concentration in apical, basolateral and cell adds up to approximately 100%.of the exposure concentration. However, at higher concentrations of CuO NMs and CuSO_4_ it was not possible to recover all copper. It is possible that the increase in copper concentration activates the synthesis of thiol containing proteins including metalothionein [74, 75] and an indiscriminate binding of copper to the thiol group may occur leading to reduction in detectable copper ion in the apical, basolateral and cell compartments. In addition, the copper may have bound to plastic used in the experiment [76, 77] (e.g. cell culture plates and the insert polycarbonate membrane) to prevent its detection. Other possible point of loss of copper include washing of the cell monolayer with PBS after aspiration of cell medium before cell digestion.

The P_app_ is frequently used to predict translocation of orally absorbed pharmaceuticals and xenobiotics, especially in drug discovery [55]. The mechanism of translocation is likely to be via passive diffusion if the P_app_ value did not change with increasing concentration of the test substance in the apical compartment [59]. P_app_ values less than 1 × 10^−6^ cm/s are an indication of malabsorption in drug development while P_app_ values greater than 10 × 10^−6^ cm/s represent good absorption [78].

In this study, the P_app_ values of differentiated Caco-2 cells treated with all concentrations of CuO NMs and CuSO_4_ for 24 h were less than 1 × 10^−6^ cm/s. In addition, at 48 h, only those cells treated with 6.34 and 12.68 μg/cm^2^ CuSO_4_ and 12.68 μg/cm^2^ of CuO NMs were greater than 1 × 10^−6^ cm/s but less than 10 × 10^−6^ cm/s. The P_app_ value at 24 and 48 h were higher than P_app_ value of mannitol (~5.0 × 10^−7^ cm/s) on differentiated Caco-2 cells [45]. This suggests that both CuO NMs and CuSO_4_ are generally malabsorbed (i.e. are not translocating across the intestinal barrier) at lower concentrations at 24 h post exposure. Therefore, it could be inferred that translocation of CuO NMs or CuSO_4_, requires tight junction stress and dysfunction.

### IL-8 production

CuO NMs and CuSO_4_ stimulated a concentration and time dependent increase in IL-8 protein production in this study in both undifferentiated and differentiated Caco-2 cells. A wide range of cell types produce interleukin 8 (IL-8), a member of the chemokine superfamily whose primary function is to mediate the activation and migration of neutrophils from peripheral blood to tissues [79]. IL-8 is involved in initiation and amplification of inflammatory processes in response to pathogenic invasion, tumour necrosis factor, cellular stress and NMs [79–81]. Production of IL-8 was much greater in undifferentiated Caco-2 cells compared to differentiated Caco-2 cells at the same concentration of Cu in the both models, which is similar to the findings of Gerloff et al. [50] who investigated the toxicity of SiO_2_ and ZnO NMs. This could be attributed to the more robust nature of differentiated Caco-2 cells as they possess most of the characteristics of human intestinal enterocytes [43]. The lower level of IL-8 production in differentiated Caco-2 cells did not appear to be a consequence of a loss of cell viability in our study. Indeed, for the controls (exposed to cell culture medium) there were higher numbers of cells observed for differentiated cells than undifferentiated cells. Despite this, there was less IL-8 production by control differentiated cells, when compared to that observed for undifferentiated cells. Our findings suggest that toxicity may be over-estimated if undifferentiated cells are used in isolation to investigate NMs toxicity. Therefore, differentiated Caco-2 cells are perhaps more appropriate for toxicity studies, when investigating cytokine production.

Another striking observation is that a peak production of IL-8 was observed at 6.34 μg/cm^2^ Cu with a decrease in production observed at 12.68 μg/cm^2^ Cu compared to the lower concentration. The peak IL-8 production was observed at the BMD 20 concentration with the decreased IL-8 production at higher concentration likely the consequences of cell death. IL-8 in the control and basolateral compartment fell below the detection limit (31.3 pg/ml), as observed by others (e.g. [51].

### NM physico-chemical properties and toxicity

NM physicochemical properties such as particle size and size distribution, agglomeration state, shape, crystal structure, chemical composition, surface area, surface chemistry, surface charge, and porosity influence the toxicity of NMs [26, 82]. The primary particle size of the CuO NMs investigated in this study is ~10 nm [25]. Immediately following dispersion in biological medium, the hydrodynamic diameter of the NMs was ~157 nm, which suggested that the NMs were agglomerated. Existing studies have also demonstrated that the dispersion medium can impact on the physico-chemical properties of NMs [82, 83]. Following incubation at 37 °C for 24 h, the hydrodynamic diameter of the NMs suspension was ~24 nm. This suggests that the NMs may become less agglomerated and/or dissolve over time.

The dissolution of CuO NMs was measured in cell culture medium in this study, and has been previously quantified in Gamble’s solution [25]. The observed level of dissociated Cu^2+^ from CuO NMs dispersed in MEM (47.79 and 53.53% at 1 and 24 h respectively) and DMEM (59.91 and 67.41% at 1 and 24 h respectively) analysed using ICP-OES and similarity in behaviour of CuSO_4_ and CuO NMs in all the studies performed suggest that the effect observed by CuO NMs are ion mediated. However, there could be some effect exerted by the particle form of copper oxide. The dissolution of CuO NMs over time has also been observed for spherical, rod and spindle-shaped platelet CuO NMs using 51, 48 and 61 mg/L respectively in complete serum free cell culture medium after 20 h incubation [84].

## Conclusion

This results of this study demonstrate that both CuO NMs and CuSO_4_ impact to a similar degree on the TEER, cell morphology, tight junction integrity, translocation and IL-8 production of differentiated Caco-2 cells in vitro. The comparable results between CuO NMs and CuSO_4_suggest that NMs induced effects is at least in part, ion mediated. Importantly, CuO NMs are no more potent than the CuSO_4_, which is important for risk assessment considerations.

Our data demonstrate that whilst more expensive and time intensive the differentiated Caco-2 model should be prioritised over undifferentiated model when assessing the impacts of NMs on the GI tract. In future studies, it is recommended that differentiated cells be used in the first instance to screen the cytotoxicity of NMs in order to rapidly provide information on their toxic potency and to identify sub-lethal concentrations to test in more comprehensive studies, which investigate the mechanism of toxicity. Future studies will also need to use more complicated in vitro intestinal models (e.g. that incorporate mucus secreting cells, inflammatory cells and M cells) to test a wider panel of NMs to identify the most appropriate model for in-depth study of NMs toxicity to GI tract.

## Additional files

Additional file 1: CuO NM dissolution study. (DOCX 12 kb)

Additional file 2: ZO-1 staining of undifferentiated Caco-2 cells. (DOCX 1046 kb)

## Abbreviations

BMD: Benchmark dose-response
CuO NMs: Copper oxide nanomaterials
CuSO_4_: Copper sulphate salt
DLS: Dynamic Light Scattering
ELISA: Enzyme-linked immunosorbent assay
FBS: Fetal bovine serum
ICP-OES: Inductive Coupled Plasma Optical Emission Spectrometry
IL-8: Interleukin-8
PBS: Phosphate buffered saline
TEER: Transepithelial electrical potential
TEM: Transmission electron microscopy

## References

[1] Desai V, Kaler SG (2008). Role of copper in human neurological disorders1–3. Am J Clin Nutr.

[2] Araya M, Olivares M, Pizarro F, González M, Speisky H, Uauy R (2003). Gastrointestinal symptoms and blood indicators of copper load in apparently healthy adults undergoing controlled copper exposure. Am J Clin Nutr.

[3] Erickson KL, Medina EA, Hubbard NE (2000). Micronutrients and Innate Immunity. J Infect Dis.

[4] Muñoz C, López M, Olivares M, Pizarro F, Arredondo M, Araya M (2005). Differential response of interleukin-2 production to chronic copper supplementation in healthy humans. Eur Cytokine Netw.

[5] Kaler SG (1998). Metabolic and molecular bases of Menkes disease and occipital horn syndrome. Pediatr Dev Pathol.

[6] Gaggelli E, Kozlowski H, Valensin D, Valensin G (2006). Copper homeostasis and neurodegenerative disorders (Alzheimer’s, prion, and Parkinson’s diseases and amyotrophic lateral sclerosis). Chem Rev.

[7] Ellingsen DG, Horn N, JAN A (2007). CHAPTER 26 - Copper. Handbook on the Toxicology of Metals.

[8] Gotteland M, Araya M, Pizarro F, Olivares M (2001). Effect of acute copper exposure on gastrointestinal permeability in healthy volunteers. Dig Dis Sci.

[9] Bouwmeester H, Dekkers S, Noordam MY, Hagens WI, Bulder AS, de Heer C, ten Voorde SECG, Wijnhoven SWP, Marvin HJP, Sips AJAM (2009). Review of health safety aspects of nanotechnologies in food production. Regul Toxicol Pharmacol.

[10] Aueviriyavit S, Phummiratch D, Maniratanachote R (2014). Mechanistic study on the biological effects of silver and gold nanoparticles in Caco-2 cells--induction of the Nrf2/HO-1 pathway by high concentrations of silver nanoparticles. Toxicol Lett.

[11] Gabbay J (2006). Copper oxide impregnated textiles with potent biocidal activities. J Ind Text.

[12] Ren G, Hu D, Cheng EW, Vargas-Reus MA, Reip P, Allaker RP (2009). Characterisation of copper oxide nanoparticles for antimicrobial applications. Int J Antimicrob Agents.

[13] Aruoja V, Dubourguier H-C, Kasemets K, Kahru A (2009). Toxicity of nanoparticles of CuO, ZnO and TiO2 to microalgae Pseudokirchneriella subcapitata. Sci Total Environ.

[14] Longano D, Ditaranto N, Cioffi N, Di Niso F, Sibillano T, Ancona A, Conte A, Del Nobile MA, Sabbatini L, Torsi L (2012). Analytical characterization of laser-generated copper nanoparticles for antibacterial composite food packaging. Anal Bioanal Chem.

[15] Civardi C, Schubert M, Fey A, Wick P, Schwarze FWMR (2015). Micronized copper wood preservatives: efficacy of ion, Nano, and bulk copper against the Brown rot fungus Rhodonia placenta. PLoS One.

[16] Soltani A, Vahed BK, Mardoukhi A, Mäntysalo M (2016). Laser sintering of copper nanoparticles on top of silicon substrates. Nanotechnology.

[17] Chang H, Jwo CS, Lo CH, Tsung TT, Kao MJ, Lin HM (2005). Rheology of CuO nanoparticle suspension prepared by ASNSS. Rev AdvMater sci.

[18] Cheng CW, Chen JK (2016). Femtosecond laser sintering of copper nanoparticles. Applied Physics A.

[19] Zenou M, Ermak O, Saar A, Kotler Z (2014). Laser sintering of copper nanoparticles. J Phys D Appl Phys.

[20] Ahamed M, Alhadlaq HA, Khan MAM, Karuppiah P, Al-Dhabi NA (2014). Synthesis, characterization, and antimicrobial activity of copper oxide nanoparticles. J Nanomater.

[21] Sambale F, Wagner S, Stahl F, Khaydarov RR, Scheper T, Bahnemann D (2015). Investigations of the toxic effect of silver nanoparticles on mammalian cell lines. J Nanomater.

[22] Asghari S, Johari SA, Lee JH, Kim YS, Jeon YB, Choi HJ, Moon MC, Yu IJ (2012). Toxicity of various silver nanoparticles compared to silver ions in Daphnia Magna. J Nanobiotechnology.

[23] Georgantzopoulou A, Serchi T, Cambier S, Leclercq CC, Renaut J, Shao J, Kruszewski M, Lentzen E, Grysan P, Eswara S (2016). Effects of silver nanoparticles and ions on a co-culture model for the gastrointestinal epithelium. Part Fibre Toxicol.

[24] Ahamed M, Akhtar MJ, Alhadlaq HA, Alrokayan SA (2015). Assessment of the lung toxicity of copper oxide nanoparticles: current status. Nanomedicine.

[25] Gosens I, Cassee FR, Zanella M, Manodori L, Brunelli A, Costa AL, Bokkers BGH, de Jong WH, Brown D, Hristozov D, et al. Organ burden and pulmonary toxicity of nano-sized copper (II) oxide particles after short-term inhalation exposure. Nanotoxicology. 2016:1–12. 10.3109/17435390.2016.1172678 PMC4975088 27132941

[26] Karlsson HL, Cronholm P, Gustafsson J, Möller L (2008). Copper oxide nanoparticles are highly toxic: a comparison between metal oxide nanoparticles and carbon nanotubes. Chem Res Toxicol.

[27] Bondarenko O, Juganson K, Ivask A, Kasemets K, Mortimer M, Kahru A (2013). Toxicity of ag, CuO and ZnO nanoparticles to selected environmentally relevant test organisms and mammalian cells in vitro: a critical review. Arch Toxicol.

[28] Lei R, Wu C, Yang B, Ma H, Shi C, Wang Q, Wang Q, Yuan Y, Liao M (2008). Integrated metabolomic analysis of the nano-sized copper particle-induced hepatotoxicity and nephrotoxicity in rats: a rapid in vivo screening method for nanotoxicity. Toxicol Appl Pharmacol.

[29] Siddiqui MA, Alhadlaq HA, Ahmad J, Al-Khedhairy AA, Musarrat J, Ahamed M (2013). Copper oxide nanoparticles induced mitochondria mediated apoptosis in human hepatocarcinoma cells. PLoS One.

[30] Chen Z, Meng H, Xing G, Chen C, Zhao Y, Jia G, Wang T, Yuan H, Ye C, Zhao F (2006). Acute toxicological effects of copper nanoparticles in vivo. Toxicol Lett.

[31] Chen D, Zhang D, Yu JC, Chan KM (2011). Effects of Cu2O nanoparticle and CuCl2 on zebrafish larvae and a liver cell-line. Aquat Toxicol.

[32] Meng H, Chen Z, Xing G, Yuan H, Chen C, Zhao F, Zhang C, Zhao Y (2007). Ultrahigh reactivity provokes nanotoxicity: explanation of oral toxicity of nano-copper particles. Toxicol Lett.

[33] Nel A, Xia T, Mädler L, Li N (2006). Toxic potential of materials at the Nanolevel. Science.

[34] Hoet PH, Brüske-Hohlfeld I, Salata OV (2004). Nanoparticles – known and unknown health risks. J Nanobiotechnology.

[35] Takenaka S, Karg E, Roth C, Schulz H, Ziesenis A, Heinzmann U, Schramel P, Heyder J (2001). Pulmonary and systemic distribution of inhaled ultrafine silver particles in rats. Environ Health Perspect.

[36] Aitken RJ, Chaudhry MQ, Boxall AB, Hull M (2006). Manufacture and use of nanomaterials: current status in the UK and global trends. Occup Med.

[37] Peixe TS, Souza Nascimento ED, Schofield KL, Arcuri ASA, Bulcão RP (2015). Nanotoxicology and exposure in the occupational setting. Occup Dis Environ Med.

[38] Stone V, Pozzi-Mucelli S, Tran L, Aschberger K, Sabella S, Vogel U, Poland C, Balharry D, Fernandes T, Gottardo S (2014). ITS-NANO - Prioritising nanosafety research to develop a stakeholder driven intelligent testing strategy. Part Fibre Toxicol.

[39] House of the Lords (2009). Nanotechnologies and Food.

[40] Burden N, Aschberger K, Chaudhry Q, Clift MJD, Doak SH, Fowler P, Johnston H, Landsiedel R, Rowland J, Stone V. The 3Rs as a framework to support a 21st century approach for nanosafety assessment. Nano Today. 2017;12:10–13

[41] Fogh J, Fogh JM, Orfeo T (1977). One hundred and twenty-seven cultured human tumor cell lines producing tumors in nude mice. J Natl Cancer Inst.

[42] Sambuy Y, Ferruzza S, Ranaldi G, De Angelis I (2001). Intestinal cell culture models: applications in toxicology and pharmacology.

[43] Sambuy Y, De Angelis I, Ranaldi G, Scarino ML, Stammati A, Zucco F (2005). The Caco-2 cell line as a model of the intestinal barrier: influence of cell and culture-related factors on Caco-2 cell functional characteristics. Cell Biol Toxicol.

[44] Natoli M, Leoni BD, D'Agnano I, D'Onofrio M, Brandi R, Arisi I, Zucco F, Felsani A (2011). Cell growing density affects the structural and functional properties of Caco-2 differentiated monolayer. J Cell Physiol.

[45] Ferruzza S, Rossi C, Scarino ML, Sambuy Y (2012). A protocol for in situ enzyme assays to assess the differentiation of human intestinal Caco-2 cells. Toxicol In Vitro.

[46] Gerloff K, Albrecht C, Boots AW, Förster I, Schins RPF (2009). Cytotoxicity and oxidative DNA damage by nanoparticles in human intestinal Caco-2 cells. Nanotoxicology.

[47] Abbott Chalew TE, Schwab KJ (2013). Toxicity of commercially available engineered nanoparticles to Caco-2 and SW480 human intestinal epithelial cells. Cell Biol Toxicol.

[48] van der Zande M, Undas AK, Kramer E, Monopoli MP, Peters RJ, Garry D, Antunes Fernandes EC, Hendriksen PJ, Marvin HJP, Peijnenburg AA (2016). Different responses of Caco-2 and MCF-7 cells to silver nanoparticles are based on highly similar mechanisms of action. Nanotoxicology.

[49] Tarantini A, Lanceleur R, Mourot A, Lavault MT, Casterou G, Jarry G, Hogeveen K, Fessard V (2015). Toxicity, genotoxicity and proinflammatory effects of amorphous nanosilica in the human intestinal Caco-2 cell line. Toxicol in Vitro.

[50] Gerloff K, Pereira DIA, Faria N, Boots AW, Kolling J, Förster I, Albrecht C, Powell JJ, Schins RPF (2013). Influence of simulated gastrointestinal conditions on particle-induced cytotoxicity and interleukin-8 regulation in differentiated and undifferentiated Caco-2 cells. Nanotoxicology.

[51] Piret JP, Vankoningsloo S, Mejia J, Noel F, Boilan E, Lambinon F, Zouboulis CC, Masereel B, Lucas S, Saout C (2012). Differential toxicity of copper (II) oxide nanoparticles of similar hydrodynamic diameter on human differentiated intestinal Caco-2 cell monolayers is correlated in part to copper release and shape. Nanotoxicology.

[52] Ferruzza S, Rossi C, Scarino ML, Sambuy Y (2012). A protocol for differentiation of human intestinal Caco-2 cells in asymmetric serum-containing medium. Toxicol in Vitro.

[53] Fisichella M, Berenguer F, Steinmetz G, Auffan M, Rose J, Prat O (2014). Toxicity evaluation of manufactured CeO2 nanoparticles before and after alteration: combined physicochemical and whole-genome expression analysis in Caco-2 cells. BMC Genomics.

[54] Lubelska K, Misiewicz-Krzemińska I, Milczarek M, Krzysztoń-Russjan J, Anuszewska E, Modzelewska K, Wiktorska K (2012). Isothiocyanate–drug interactions in the human adenocarcinoma cell line Caco-2. Mol Cell Biochem.

[55] Hubatsch I, Ragnarsson EGE, Artursson P (2007). Determination of drug permeability and prediction of drug absorption in Caco-2 monolayers. Nat Protocols.

[56] Lefebvre DE, Venema K, Gombau L, Valerio LG, Raju J, Bondy GS, Bouwmeester H, Singh RP, Clippinger AJ, Collnot EM (2015). Utility of models of the gastrointestinal tract for assessment of the digestion and absorption of engineered nanomaterials released from food matrices. Nanotoxicology.

[57] Chen X-M, Elisia I, Kitts DD (2010). Defining conditions for the co-culture of Caco-2 and HT29-MTX cells using Taguchi design. J Pharmacol Toxicol Methods.

[58] Beloqui A, des Rieux A, Préat V. Mechanisms of transport of polymeric and lipidic nanoparticles across the intestinal barrier. Adv Drug Deliv Rev. 2016;106(Part B):242–255. 10.1016/j.addr.2016.04.014 27117710

[59] Chen G, Lianqin Z, Fenghua Z, Fang Z, Mingming S, Kai H (2015). Comparative evaluation of nano-CuO crossing Caco-2 cell monolayers and cellular uptake. J Nanopart Res.

[60] Chang H-J, Choi S-W, Ko S-H, Chun H-S (2011). Effect of particle size of zinc oxides on cytotoxicity and cell permeability in Caco-2 cells. J Food Sci Nutr.

[61] Schleh C, Semmler-Behnke M, Lipka J, Wenk A, Hirn S, Schäffler M, Schmid G, Simon U, Kreyling WG (2012). Size and surface charge of gold nanoparticles determine absorption across intestinal barriers and accumulation in secondary target organs after oral administration. Nanotoxicology.

[62] Jacobsen NR, Pojano G, Wallin H, Jensen KA. Nanomaterial dispersion protocol for toxicological studies in ENPRA. Internal ENPRA Project Report. The National 885 Research Centre for the Working Environment 2010.

[63] Horobin RW (2011). How Romanowsky stains work and why they remain valuable — including a proposed universal Romanowsky staining mechanism and a rational troubleshooting scheme. Biotech Histochem.

[64] Bolea E, Jimenez-Lamana J, Laborda F, Abad-Alvaro I, Blade C, Arola L, Castillo JR (2014). Detection and characterization of silver nanoparticles and dissolved species of silver in culture medium and cells by AsFlFFF-UV-vis-ICPMS: application to nanotoxicity tests. Analyst.

[65] des Rieux A, Fievez V, Theate I, Mast J, Preat V, Schneider YJ. An improved in vitro model of human intestinal follicle-associated epithelium to study nanoparticle transport by M cells. Eur J Pharm Sci. 2007;30(5):380–391. 10.1016/j.ejps.2006.12.006 17291730

[66] Han X, Gelein R, Corson N, Wade-Mercer P, Jiang J, Biswas P, Finkelstein JN, Elder A, Oberdörster G: Validation of an LDH assay for assessing nanoparticle toxicity. Toxicology 2011, 287(0):99–104. 10.1016/j.tox.2011.06.011 PMC4070602 21722700

[67] Stone V, Johnston HJ, Balharry D, Gernand JM, Gulumian M (2016). Approaches to develop alternative testing strategies to inform human health risk assessment of nanomaterials. Risk Anal.

[68] Chen C-C, Chen JJ (2014). Benchmark dose calculation for ordered categorical responses. Risk Anal.

[69] Bouwmeester H, Poortman J, Peters RJ, Wijma E, Kramer E, Makama S, Puspitaninganindita K, Marvin HJP, Peijnenburg AACM, Hendriksen PJM (2011). Characterization of translocation of silver nanoparticles and effects on whole-genome gene expression using an in vitro intestinal epithelium Coculture model. ACS Nano.

[70] Nishitani Y, Zhang L, Yoshida M, Azuma T, Kanazawa K, Hashimoto T, Mizuno M (2013). Intestinal anti-inflammatory activity of lentinan: influence on IL-8 and TNFR1 expression in intestinal epithelial cells. PLoS One.

[71] Walczak AP, Hendriksen PJM, Woutersen RA, van der Zande M, Undas AK, Helsdingen R, van den Berg HHJ, Rietjens IMCM, Bouwmeester H (2015). Bioavailability and biodistribution of differently charged polystyrene nanoparticles upon oral exposure in rats. J Nanopart Res.

[72] Walczak AP, Kramer E, Hendriksen PJM, Tromp P, Helsper JPFG, van der Zande M, Rietjens IMCM, Bouwmeester H (2015). Translocation of differently sized and charged polystyrene nanoparticles in in vitro intestinal cell models of increasing complexity. Nanotoxicology.

[73] Bellmann S, Carlander D, Fasano A, Momcilovic D, Scimeca JA, Waldman WJ, Gombau L, Tsytsikova L, Canady R, Pereira DI (2015). Mammalian gastrointestinal tract parameters modulating the integrity, surface properties, and absorption of food-relevant nanomaterials. Wiley Interdiscip Rev Nanomed Nanobiotechnol.

[74] Gaetke LM, Chow-Johnson HS, Chow CK (2014). Copper: toxicological relevance and mechanisms. Arch Toxicol.

[75] Letelier ME, Lepe AM, Faúndez M, Salazar J, Marín R, Aracena P, Speisky H (2005). Possible mechanisms underlying copper-induced damage in biological membranes leading to cellular toxicity. Chem Biol Interact.

[76] Malysheva A, Ivask A, Hager C, Brunetti G, Marzouk ER, Lombi E, Voelcker NH (2016). Sorption of silver nanoparticles to laboratory plastic during (eco)toxicological testing. Nanotoxicology.

[77] Sekine R, Khurana K, Vasilev K, Lombi E, Donner E (2015). Quantifying the adsorption of ionic silver and functionalized nanoparticles during ecotoxicity testing: test container effects and recommendations. Nanotoxicology.

[78] Yee S (1997). In vitro permeability across Caco-2 cells (colonic) can predict in vivo (small intestinal) absorption in man—fact or myth. Pharm Res.

[79] Puthothu B, Krueger M, Heinze J, Forster J, Heinzmann A (2006). Impact of IL8 and IL8-receptor alpha polymorphisms on the genetics of bronchial asthma and severe RSV infections. Clin Mol Allergy.

[80] Hoffmann E, Dittrich-Breiholz O, Holtmann H, Kracht M (2002). Multiple control of interleukin-8 gene expression. J Leukoc Biol.

[81] Kermanizadeh A, Pojana G, Gaiser BK, Birkedal R, Bilanicova D, Wallin H, Jensen KA, Sellergren B, Hutchison GR, Marcomini A (2013). In vitro assessment of engineered nanomaterials using a hepatocyte cell line: cytotoxicity, pro-inflammatory cytokines and functional markers. Nanotoxicology.

[82] Oberdörster G, Maynard A, Donaldson K, Castranova V, Fitzpatrick J, Ausman K, Carter J, Karn B, Kreyling W, Lai D (2005). Principles for characterizing the potential human health effects from exposure to nanomaterials: elements of a screening strategy. Part Fibre Toxicol.

[83] Kang T, Guan R, Chen X, Song Y, Jiang H, Zhao J (2013). In vitro toxicity of different-sized ZnO nanoparticles in Caco-2 cells. Nanoscale Res Lett.

[84] Misra SK, Nuseibeh S, Dybowska A, Berhanu D, Tetley TD, Valsami-Jones E (2014). Comparative study using spheres, rods and spindle-shaped nanoplatelets on dispersion stability, dissolution and toxicity of CuO nanomaterials. Nanotoxicology.

