# Supplementary material for: Impact of copper oxide nanomaterials on differentiated and undifferentiated Caco-2 intestinal epithelial cells; assessment of cytotoxicity, barrier integrity, cytokine production and nanomaterial penetration
Source: Part Fibre Toxicol. 2017 Aug 23;14:31. doi: 10.1186/s12989-017-0211-7 (PMC5569458; doi:10.1186/s12989-017-0211-7)
Supplement: Supplementary file 1 — CuO NM dissolution study. (DOCX 12 kb) [file 12989_2017_211_MOESM1_ESM.docx]

**CuO NM dissolution study**

A concentration of 10 mg/ml of Cu stock suspension of CuO NMs, which was prepared in phosphate buffer was diluted in MEM or DMEM supplemented with 20 % FBS and 1% Pen/Strep to obtain a concentration of 50 µg/ml (working stock). Part of the suspension was ultra-filtrated with 10kDa Amicon centrifugal filters (30 min, 5000 RPM), weighting 15g as feed suspension. One millilitre of HNO_3_ ultrapure (70%) was added to 10g of filtered solution and sample collected for ICP-OES analysis. As well, 1 mL of HNO_3_ was added to 10 g of feed suspension to mineralize all the content of Cu present in solid form the suspension was centrifuged using an Ultra-centrifugal Filter (UCF) unit (Amicon ultra-15, 10 kDa, Millipore) at 5000 rpm for 30 min. An aliquot of 10 ml of the filtered solvent containing the dissolved Cu^2+^ ions was subjected to elemental analysis using ICP-OES.

**Table 1.** Cu^2+^/CuO weight ratio percentage of CuO NMs dispersed in DMEM and MEM. The filtered solvent containing the dissolved Cu^2+^ ions was subjected to elemental analysis using ICP-OES.

| Time (h) | MEM (Cu^2+^/CuO %) | DMEM (Cu^2+^/CuO %) |
| --- | --- | --- |
| 1 | 49.79 | 53.53 |
| 24 | 59.91 | 67.41 |
