# Supplementary material for: Impact of copper oxide nanomaterials on differentiated and undifferentiated Caco-2 intestinal epithelial cells; assessment of cytotoxicity, barrier integrity, cytokine production and nanomaterial penetration
Source: Part Fibre Toxicol. 2017 Aug 23;14:31. doi: 10.1186/s12989-017-0211-7 (PMC5569458; doi:10.1186/s12989-017-0211-7)
Supplement: Supplementary file 2 — ZO-1 staining of undifferentiated Caco-2 cells. (DOCX 1046 kb) [file 12989_2017_211_MOESM2_ESM.docx]

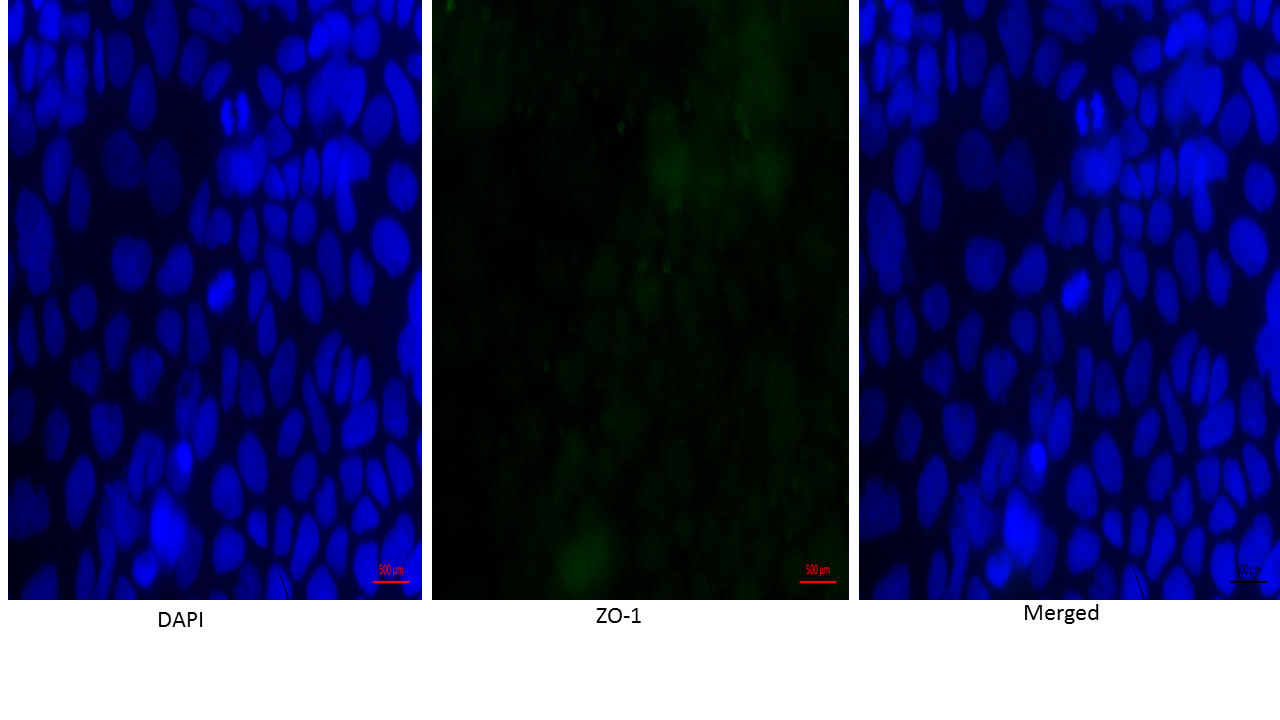


ZO-1 staining of undifferentiated Caco-2 cells. Cells (3.13 x 10^5^ cells/cm^2^) were grown on a glass coverslip in a 24 well plate (Coaster Corning, Flintshire, UK). After 24 h of incubation, the cells were fixed and stained for the tight junction protein ZO-1 (green) and nucleus with DAPI (blue). The images were obtained with Zeiss fluorescence microscopy equipped with AxioCam camera. Scale bar = 500 µm.
